# Massive expression of germ cell-specific genes is a hallmark of cancer and a potential target for novel treatment development

**DOI:** 10.1038/s41388-018-0357-2

**Published:** 2018-06-15

**Authors:** Jan Willem Bruggeman, Jan Koster, Paul Lodder, Sjoerd Repping, Geert Hamer

**Affiliations:** 1 Center for Reproductive Medicine, Amsterdam Research Institute Reproduction and Development, Academic Medical Center, University of Amsterdam, Amsterdam, The Netherlands; 2 Department of Oncogenomics, Academic Medical Center, University of Amsterdam, Amsterdam, The Netherlands

## Abstract

Cancer cells have been found to frequently express genes that are normally restricted to the testis, often referred to as cancer/testis (CT) antigens or genes. Because germ cell-specific antigens are not recognized as “self” by the innate immune system, CT-genes have previously been suggested as ideal candidate targets for cancer therapy. The use of CT-genes in cancer therapy has thus far been unsuccessful, most likely because their identification has relied on gene expression in whole testis, including the testicular somatic cells, precluding the detection of true germ cell-specific genes. By comparing the transcriptomes of micro-dissected germ cell subtypes, representing the main developmental stages of human spermatogenesis, with the publicly accessible transcriptomes of 2617 samples from 49 different healthy somatic tissues and 9232 samples from 33 tumor types, we here discover hundreds of true germ cell-specific cancer expressed genes. Strikingly, we found these germ cell cancer genes (GC-genes) to be widely expressed in all analyzed tumors. Many GC-genes appeared to be involved in processes that are likely to actively promote tumor viability, proliferation and metastasis. Targeting these true GC-genes thus has the potential to inhibit tumor growth with infertility being the only possible side effect. Moreover, we identified a subset of GC-genes that are not expressed in spermatogonial stem cells. Targeting of this GC-gene subset is predicted to only lead to temporary infertility, as untargeted spermatogonial stem cells can recover spermatogenesis after treatment. Our GC-gene dataset enables improved understanding of tumor biology and provides multiple novel targets for cancer treatment.

## Introduction

Many genes have been identified that drive the transition process of healthy cells into cancer cells. Such oncogenes contribute to the acquisition of cancer-specific hallmarks [1, 2], such as uncontrolled cell divisions, genome instability, angiogenesis, aberrant apoptosis regulation, and telomere maintenance. Targeting these hallmark processes is effectively used by many current cancer therapies. However, because the majority of these processes are also widely used by non-cancerous cells, these therapies most often cause severe side effects. These side effects could theoretically be avoided by targeting genes that are almost exclusively expressed in cancer cells. One group of genes that is currently studied to achieve this goal are the genes encoding cancer/testis antigens (CT-genes); defined as proteins that are exclusively present in tumors and adult gametogenic tissues [3, 4]. Targeting these proteins potentially has only one side effect, namely infertility. Moreover, male germ cells are unique because they do not express genes encoding the HLA class I transmembrane protein complexes that the immune system uses to differentiate ‘self’ from ‘non-self’ (invading) cells [5]. The ectopic presentation of germ cell-specific antigens on these HLA complexes in cancer cells may therefore not be recognized as ‘self’ by cytotoxic T cells, leading to an immune response that is highly cancer-specific. This characteristic theoretically makes these antigens ideal candidate targets for immunotherapy [6].

In addition, identification and analysis of true germ cell-specific genes will help us to better understand the mechanisms that drive tumor development and function. Some germ cell-specific genes may just be accidentally expressed in tumors due to a “randomly” disturbed regulation of gene expression [3, 4]. However, during gametogenesis, many germ cell-specific genes regulate processes like chromosome pairing, gene expression, DNA damage repair and cell cycle checkpoints [7], and are thus potential active drivers of tumorigenesis. Indeed, recent studies have shown that expression of several germ cell-specific genes actively contributes to both tumor development and survival [8, 9].

Unfortunately, most CT-genes have been identified by comparing gene expression data of various tumor types and healthy tissues with whole testis [9–14]. However, the germ cells represent only a fraction of all testicular cells, and whole testis also includes many somatic cells. These include the testis-specific endocrine Leydig and Sertoli cells, but also generic cell types, such as epithelial, smooth muscle, and blood cells. Targeting of gene products expressed in these somatic cell types is thus likely to affect healthy tissues outside the testis or, in case of Leydig and Sertoli cells, may have unknown long-term endocrine side effects.

With the aim to identify true germ cell-specific cancer genes (GC-genes), we here used the transcriptomes of specific germ cell-types present in healthy human spermatogenesis, which we recently unraveled in our laboratory [15]. We compared this dataset with data from the Genotype-Tissue Expression (GTEx) project [16] and The Cancer Genome Atlas (TCGA) [17] to achieve tumor specificity of germ cell gene expression. Moreover, we used germ cell-type-specific gene expression data to exclude genes expressed in spermatogonial stem cells. Because spermatogonial stem cells can reinitiate and maintain life-long spermatogenesis, targeting of the remaining gene products would only lead to temporal infertility and would thus, in principle, have no long-term side effects. Using this bioinformatic approach, we identified and characterized 756 germ cell-specific genes that are widely expressed in cancer.

## Results and discussion

Where discovery of most CT-genes depended on whole testis expression data, we here used a unique list of 16 589 genes expressed in human male germ cells generated in our laboratory [15] to identify true germ cell-specific cancer genes (GC-genes). This germ cell transcriptome has been generated from tissue samples provided by 6 men who underwent vasectomy reversal procedures. Specific germ cell subtypes were isolated using laser capture microdissection, and consequentially sequenced using Illumina HiSeq 2000, as described in detail in Jan et al. [15]. Using R2, a genomics analysis and visualization platform we developed recently [18], we compared this germ cell subtype specific transcriptome [15] to data from the Genotype-Tissue Expression (GTEx) project [16] and The Cancer Genome Atlas (TCGA) [17] (Supplementary Data 1A). The GTEx and TCGA datasets were both downloaded in November 2015 from the GTEx data portal (GTEx_Analysis_V4_RNA-seq_RNA-SeQCv1.1.8_gene_rpkm.gct.gz, RSEM processed [16]) and TCGA data portal (unc.edu_****.IlluminaHiSeq_RNASeqV2.Level_3.1.*.0.tar.gz, RSEM processed [17]), respectively. Analyses were performed by custom PERL scripts, or R/Bioconductor. Images were generated in R/Bioconductor or R2 platform (r2.amc.nl).

**Fig. 1:**
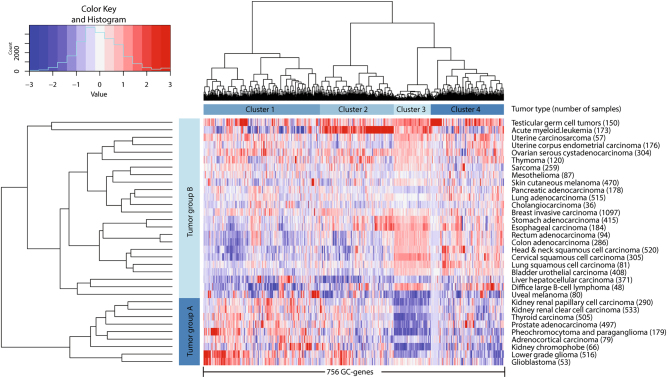
Hundreds of germ cell-specific genes are widely expressed in tumors. Shown here as hierarchical clustering of the average expression per tumor group (Euclidean distance, ward linkage). These germ cell cancer genes (GC-genes) divide tumors in two main groups, mainly based on GC-gene cluster 3, containing genes involved in mitotic and meiotic metaphase regulation. Gene expression levels are indicated by a *Z*-score-dependent color, where blue and red represent low and high expression, respectively

At the moment of our analysis, these databases contained 2617 samples from 53 different healthy somatic tissues (Supplementary Data 1B) and 9232 samples from 33 tumor types (Supplementary Data 1C), respectively. From the GTEx dataset, we excluded ovary and testis tissues. In addition, we excluded transformed lymphocytes and transformed fibroblasts, as they are transformed cell lines instead of whole tissues and may have upregulated genes associated with cancer cells. For each of the three datasets, the maximum expression measured per gene was used to determine arbitrary inclusion criteria that remove background noise (from the expression in male germ cells and tumors) and avoid false-positive GC-genes (expression in healthy somatic tissues) (Supplementary Figure 1). We have been most strict on the GTEx database (only 13% included) to avoid false-positives. Our comparison yielded 756 putative novel GC-genes that are highly expressed in germ cells, not expressed in any somatic tissue and highly expressed in tumors (Supplementary Data 1D). In order to visualize how the 756 GC-genes vary by tumor type, we stratified their expression in 33 tumor types in a heat map, showing that hundreds of GC-genes are expressed in all tumor types (Fig. 1).

In order to enable everybody to determine their own inclusion criteria, we have developed a web-based application. For all interactively chosen cut-off criteria, the consequent GC-gene list can be downloaded. This application is available from: https://www.amsterdamresearch.org/web/reproduction-and-development/tools.htm.

**Fig. 2:**
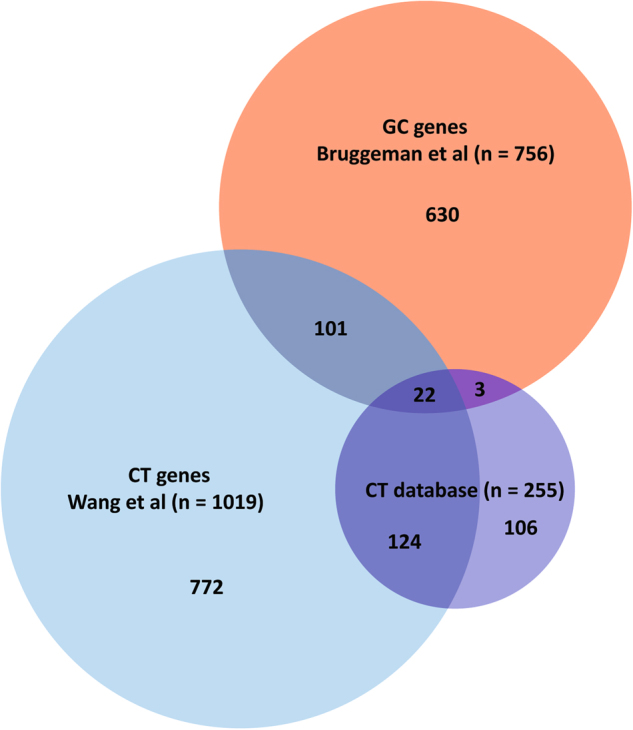
Most GC-genes have not been described before as CT-gene. Venn diagram comparing the present analysis of germ cell-specific cancer (GC) genes (red) to earlier identified Cancer/Testis (CT) genes by Wang et al. (light blue) and the CT-database (dark blue). The number in each section represents the number of genes. The overlap between the CT database [10], Wang et al. [9], and the present analysis was assessed by converting gene names to one common annotation (Supplementary Data 10A–C). 21 out of 276 genes in the CT database were either merged with existing genes (*n* = 19) or could not be retrieved (*n* = 2) (Supplementary Data 10D)

To avoid false-positive results, the selection criteria we applied to identify GC-genes are more stringent than previous selection criteria used to identify CT-genes. Moreover, whereas most studies allowed expression in 1–2 tissues other than the testis [11–14], our selection excludes all genes expressed in healthy tissues other than the testis. Thus, for most of the 756 genes identified in this study we can be certain that they are true GC-genes. However, lowly expressed genes that are only shortly or temporarily expressed, are only expressed in rare cell types, or only expressed under certain conditions may have escaped our selection. In addition, because germ cell tumors can be expected to express many germ cell-specific genes, we analyzed which genes would not have been included in our initial list after excluding testicular germ cell tumors, and identified a subset of 45 GC-genes that are only expressed in germ cell tumors (Supplementary Data 2). From our original list of 16,589 genes expressed in male germ cells, 165 genes are present in a database containing genes specifically expressed in cancer and whole testis tissue: the CT database [10]. From the 255 CT-genes present in this database at the moment of our analysis, only 25 overlap with our newly identified 756 GC-genes. This can be explained by the fact that the testis for a large part consists of somatic cells. Germ cell-specific RNAs can therefore be diluted below detection levels in whole testis lysates, while testicular somatic genes are not included in our analysis. Indeed, from a more recent analysis that revealed 1019 potential CT-genes [9], only 123 (12%) were also present in our analysis (Fig. 2). In addition, the interactive Venn diagram can be used to show that being less strict on high tumor expression has the most significant impact on the overlap with the CT-gene studies. This implies that many genes identified by the previous CT-gene studies were not highly expressed in at least one of the tumor types that we investigated. These data combined, our current analysis has identified 630 GC-genes that have not been previously identified as CT-genes, of which 615 are expressed in non-testicular tumors.

Hierarchical cluster analysis revealed that, based on expression of GC-genes, the tumors form two main groups, mostly characterized by high or low expression of a specific subset of GC-genes (gene cluster 3) (Fig. 1 and Supplementary Data 3A). We have investigated the biological processes of this gene cluster using DAVID Bioinformatics Resources 6.7 [19], and reported only significant annotation clusters (enrichment score > 1.3, corresponding to *p* < 0.05). Interestingly, this gene ontology (GO) analysis revealed that gene cluster 3 predominately contains genes involved in M-phase and cell cycle regulation, intriguingly both mitotic and meiotic (Table 1). Also processes pivotal to meiosis, such as DNA double-strand break repair and homologous recombination, are well represented in this cluster (Supplementary Data 3D). Further GO-analysis revealed that six biological processes are significantly represented by all 756 GC-genes (Supplementary Data 3F): the regulation of transcription and gene expression, including the metabolic processes required for RNA and DNA synthesis, the M-phase of the mitotic and meiotic cell cycle, DNA double-stranded break repair, DNA metabolic processes, spermatogenesis, and cell adhesion. Additional GO-analysis was performed on the top 25% GC-genes that were most widely expressed in tumors. Six biological processes appeared to be significantly represented by these 189 GC-genes, including cell cycle regulation and checkpoints, post-translational protein modification and DNA damage responses (Supplementary Data 3G). In line with previous research [8, 9, 20], these processes suggests that GC-genes are not just randomly expressed germ cell-specific genes but may actually contribute to tumor cell survival, proliferation, and metastasis.

**Table 1:** GC-genes represent processes that are likely to contribute to tumor cell survival, proliferation, and metastasis

Set (suppl. info)	GO-term	Description	Enrichment
All GC-genes (3F)	GO:0006139	Nucleobase, nucleoside, nucleotide, and nucleic acid metabolic process	14.21
	GO:0000279	M-phase	5.73
	GO:0006302	Double-strand break repair	3.04
	GO:0006259	DNA metabolic process	2.70
	GO:0048610	Reproductive cellular process	2.50
	GO:0007156	Homophilic cell adhesion	1.55
25% most widely expressed in cancer (3G)	GO:0044260	Cellular macromolecule metabolic process	3.90
	GO:0022403	Cell cycle phase	3.59
	GO:0032446	Protein modification by small protein conjugation	1.78
	GO:0006974	Response to DNA damage stimulus	1.51
	GO:0007017	Microtubule-based process	1.44
	GO:0000075	Cell cycle checkpoint	1.41
Cluster 1 (3B)^a^	GO:0007156	Homophilic cell adhesion	4.70
	GO:0006350	Transcription	3.97
	GO:0016339	Calcium-dependent cell–cell adhesion	1.73
	GO:0050953	Sensory perception of light stimulus	1.62
	GO:0001539	Ciliary or flagellar motility	1.39
Cluster 2 (3C)^a^	GO:0006350	Transcription	19.47
Cluster 3 (3D)^a^	GO:0000279	M-phase (mitosis)	18.63
	GO:0000279	M-phase (meiosis)	16.60
	GO:0006259	DNA metabolic process	5.41
	GO:0010564	Regulation of cell cycle process	4.47
	GO:0007017	Microtubule-based process	4.47
	GO:0006310	DNA recombination	4.32
	GO:0033043	Regulation of organelle organization	2.72
	GO:0000075	Cell cycle checkpoint	2.38
Cluster 4 (3E)^a^	GO:0051327	M-phase of meiotic cell cycle	1.97
	GO:0019953	Sexual reproduction	1.71
	GO:0043046	DNA methylation during gametogenesis	1.61
GC-genes encoding cell surface proteins (4B)	GO:0051239	Regulation of multicellular organismal process^b^	1.78
	GO:0051239	Regulation of multicellular organismal process^b^	1.58
	GO:0006836	Neurotransmitter transport	1.49
GC-genes not detected in whole testis (5B)	GO:0045449	Regulation of transcription	8.07
	GO:0007156	Homophilic cell adhesion	3.04
	GO:0045494	Photoreceptor cell maintenance	1.45
Cancer-specific genes that are not GC-genes (7B)	GO:0006955	Immune response	2.24

Summary of gene ontology (GO) analysis of GC-genes. Enrichment equals −log_10_(*p*), where 1.3 is equivalent to *p* = 0.05 and *p* represents the geometric mean of *p*-values in an annotation cluster. Only a description of the first term of each statistically significant (enrichment > 1.3) annotation cluster is shown. Full results are shown in corresponding supplementary data for each subset (3B–G, 4B, 5B, and 7B)

^a^As referred to in Fig. 1

^b^These annotation clusters are not exactly the same, but both include the same GO-term, which has the lowest *p*-value in each cluster

Because proteins that are located on the outer cell surface would be ideal targets for induced adaptive immune (therapeutic) responses, we used the Panther 10.0 classification system [21] and identified 17 GC-genes that are known to encode cell surface proteins (Table 2 and Supplementary Data 4A). These genes are predominantly responsible for the regulation of multicellular organismal processes, cell proliferation and cell–cell communication (Supplementary Data 4B). Interestingly, all tumors express at least one of these 17 genes. Because CT-genes have previously been identified using gene expression profiles of whole testis, including the testicular somatic cells, we additionally compared gene expression of whole testis tissue from the GTEx project [16] to gene expression in human male germ cells [15]. For each gene with high expression (>1.6) in male germ cells [15] and low expression (<1.8) in any other tissue [16], the difference in expression compared to whole testis was calculated. In order to correct for using different expression distributions, genes with a difference in expression above one log_2_ value (i.e., double expression) were included. This resulted in a list of 706 genes that are expressed in germ cells, but were not previously detected in testis as a whole. When comparing our list of 756 GC-genes to these 706 genes, we identified a subset of 334 GC-genes (44%) whose expression is very low or undetectable (<1.8) in testis as a whole [16] (Supplementary Data 5A). Interestingly, among this subset of 334 GC-genes, the average expression across all tumors is higher than in the remaining GC-genes (*p* = 0.032, Student’s *t*-test assuming unequal variances). GO-analysis of this subset identified transcription regulation and cell adhesion as significantly enriched processes (Supplementary Data 5B), both of which essential for germ cell development, as well as tumor proliferation and metastasis. This, and the fact that they are highly germ cell and cancer specific, makes these genes interesting candidates for future research.

**Table 2:** GC-genes that are known to encode cell surface proteins

Gene ID	Max. expression^a^ in germ cells [15]	Max. expression^b^ in non-cancerous somatic tissues (GTEx v4)	Max. expression^c^ in tumors (TCGAN 2016)	Number of tumor types that show RNA expression (TCGAN 2016)
CHRNA7	1.88	1.35	7.17	1
CLEC12B	4.14	1.21	7.13	1
GP1BA	1.91	1.39	8.02	2
HMMR	6.99	1.59	9.25	25
IGLL1	6.50	0.39	8.09	2
IL12RB2	3.27	1.13	8.46	5
KCNH5	2.47	0.69	6.62	1
LRFN4	5.61	0.94	10.04	32
MPL	3.47	0.40	8.23	1
NLGN1	5.09	1.65	9.75	6
NRG1	4.44	1.40	8.40	8
PLK4	7.20	1.51	8.90	24
SLC6A2	6.75	1.04	10.83	1
TNFSF4	2.56	1.61	8.37	15
TRPV1	4.06	0.83	8.57	31
UMODL1	3.55	0.30	7.41	1
WNT7A	2.31	1.62	8.36	5

Seventeen GC-genes are known to encode cell surface proteins. Full results are shown in Supplementary Data 4A. A gene ontology analysis of these genes is available in Supplementary Data 4B

^a^Maximum expression of the gene across all stadia of germ cell development

^b^Maximum expression of the gene across all non-cancerous somatic (healthy) tissues (*n* = 49)

^c^Maximum expression of the gene across all tumor types (*n* = 33)

Previously, CT-genes have been divided in X and non-X CT-genes, depending on whether they are located on the X chromosome or not. According to the CT-database, approximately half the CT-genes are CT-X [10]. However, of the 1019 CT-genes identified by Wang et al. [9], only 105 were located on the X chromosome. In line with this study, our analysis returned only 29 (4%) X-linked GC-genes and GC-genes seem to be distributed relatively evenly across all chromosomes (Supplementary Figure 2).

To validate to what extent germ cell-specific RNA expression reflects protein expression in various human tissues we used the Human Protein Atlas (v15) [22]. From the atlas, we retrieved all proteins expressed in testis or ovary and selected for highly reliable immunohistochemistry (Premium Tissue). In addition, because many CT-genes are known to be expressed in trophoblasts [23] that will later form the placenta, we similarly retrieved all proteins expressed in placenta. The resulting proteins were then aligned with our 756 GC-genes, resulting in a list of 49 genes that were manually checked for germ cell- or placenta-specific protein expression (Supplementary Data 6). This yielded three proteins that are exclusively present in placenta and 24 proteins that are present in male germ cells and not in somatic cells of the testis or elsewhere (Table 3).

**Table 3:** GC-genes whose restricted expression in non-cancerous tissues is validated on the protein level

Gene ID	Exclusively expressed in
AKAP3	Testis
CTCFL	Testis
DMC1	Testis
DPEP3	Testis
DPPA4	Testis & placenta
FATE1	Testis
HDGFL1	Testis
HORMAD1	Testis
HSPA1L	Testis
KIAA1210	Testis
LIN28B	Placenta
LRRC37A2	Testis
LUZP4	Testis
MAGEA4	Testis & placenta
MAGEB1	Testis
PAPPA	Placenta
PAPPA2	Placenta
PRSS21	Testis
RBMXL2	Testis
RIMBP3	Testis
SGOL2	Testis
SMC1B	Testis
STK31	Testis
SUV39H2	Testis
SYCP3	Testis
TKTL1	Testis
TPTE	Testis

Twenty-seven GC-genes are exclusively expressed in the testis or placenta tissue on the protein level, according to data from the Human Protein Atlas [22]. Full results are shown in Supplementary Data 6

To develop a therapy without side effects in healthy tissues it would in principle be sufficient to identify genes that are uniquely expressed in tumors. For this, our list of human germ cell expressed genes would not be required. We therefore performed a similar analysis without our list of germ cell expressed genes and including testis from the GTEx database. This resulted in 724 cancer-specific genes, of which 301 genes appeared not to be GC-genes (Supplementary Data 7A). However, GO-analysis revealed that these 301 genes are predominately involved in immunological responses (Supplementary Data 7B). Hence, in contrast to GC-genes, targeting these genes as a cancer therapy can be expected to lead to immunological side effects.

Infertility is a major side effect of current anticancer treatments, and it would still be a potential side effect when targeting most GC-genes. A way to circumvent this would be to exclude genes expressed in the spermatogonial stem cells. In humans, these stem cells are included in the pool of quiescent or mitotically proliferating and differentiating spermatogonia, and are required to maintain life-long spermatogenesis. Because our dataset contains information about germ cell-type-specific gene expression [15], we were able to exclude genes expressed in spermatogonia. Of the 756 GC-genes, 69 displayed negligible expression in the spermatogonial stages (Supplementary Data 8). Hence, targeting these 69 GC-genes would not affect the spermatogonial stem cells and therefore only lead to temporary infertility. Importantly, we have recently found that spermatogonia already express many mRNAs that are not translated until later stages during spermatogenesis [15]. This implies that the number of GC-genes that can be targeted without inducing permanent infertility will most likely be larger than 69.

We here show that expression of hundreds of germ cell-specific genes may not only contribute to already established hallmarks of cancer [1, 2], but can be considered as a hallmark of cancer in itself. Germ cells and cancer cells share the intrinsic drive to propagate, regardless of survival of the soma [24–26]. Studying the behavior and characteristics of germ cells may thus lead to novel insights in cancer development. Because our datasets are publically available, more tumor types can now be analyzed on the expression of germ cell-specific genes. We anticipate that this will lead to a better understanding of tumor biology and improved treatment options.

## Electronic supplementary material

Supplementary data legends

Supplementary Figure 1

Supplementary Figure 2

Supplementary Dataset 1
