# Supplementary material for: Massive expression of germ cell-specific genes is a hallmark of cancer and a potential target for novel treatment development
Source: Oncogene. 2018 Jun 15;37(42):5694–700. doi: 10.1038/s41388-018-0357-2 (PMC6193945; doi:10.1038/s41388-018-0357-2)
Supplement: Supplementary file 1 — Supplementary data legends [file 41388_2018_357_MOESM1_ESM.pdf]

Supplementary data legends

One Excel (.xlsx) file (24.6 MB) contains all supplementary data:

Number	*Description*
**1A**	Combined data from expression of 16 589 genes in germ cells, 49 non-cancerous somatic tissues, and 33 tumors
**1B**	Non-cancerous somatic tissue types (n = 53)
**1C**	Abbreviations and full names of included cancer types (n = 33)
**1D**	Combined data from expression of 756 cancer-germ cell specific genes, 49 non-cancerous somatic tissues, and 33 tumors
**2**	GC-genes with high expression in testicular germ cell tumors (n = 45)
**3A**	List of 756 cancer-germ cell specific genes with associated cluster (as visualized in figure 1)
**3B**	Biological processes of GC-gene subset cluster 1 (n = 293)
**3C**	Biological processes of GC-gene subset cluster 2 (n = 186)
**3D**	Biological processes of GC-gene subset cluster 3 (n = 93)
**3E**	Biological processes of GC-gene subset cluster 4 (n = 184)
**3F**	Biological processes of 756 cancer-germ cell specific genes, 676 of which were recognized for analysis
**3G**	Biological processes of the 25% (n = 189) cancer-germ cell specific gene that were most widely expressed in cancer, 178 of which were recognized for analysis
**4A**	Putative cell surface protein encoding genes (n = 17)
**4B**	Gene ontology analysis of putative cell surface protein encoding genes (n = 17), all of which were recognized for analysis
**5A**	Cancer-germ cell specific genes that are not detected in whole testis tissue (n = 334)
**5B**	Biological processes of 334 cancer-germ cell specific genes that are not detected in whole testis tissue, 300 of which were recognized for analysis
**6**	Validation of exclusive expression on the protein level of 49 GC-genes
**7A**	List of 724 genes that are expressed in tumors but not in normal healthy tissues (comparison between GTEx and TCGA), 422 of which are GC-genes.
**7B**	Gene ontology enrichment analysis of 301 cancer-specific genes that are not GC-genes
**8**	GC-genes that are not expressed in germ cells until after the spermatogonial stage (n = 69)
**9**	List of 1526 excluded genes for which not enough information was available in the GTEx and/or TCGA database
**10A**	List of 756 GC-genes with alternative names for finding overlap with similar studies
**10B**	List of 1 019 CT-genes identified by Wang et al (2016) with alternative names for finding overlap with similar studies
**10C**	List of 255 CT-genes from the CT-database with alternative names for finding overlap with similar studies
**10D**	List of 21 CT-genes from the CT-database that have been excluded for finding overlap with similar studies
