# Supplementary material for: Massive expression of germ cell-specific genes is a hallmark of cancer and a potential target for novel treatment development
Source: Oncogene. 2018 Jun 15;37(42):5694–700. doi: 10.1038/s41388-018-0357-2 (PMC6193945; doi:10.1038/s41388-018-0357-2)
Supplement: Supplementary file 2 — Supplementary Figure 1 [file 41388_2018_357_MOESM2_ESM.pdf]

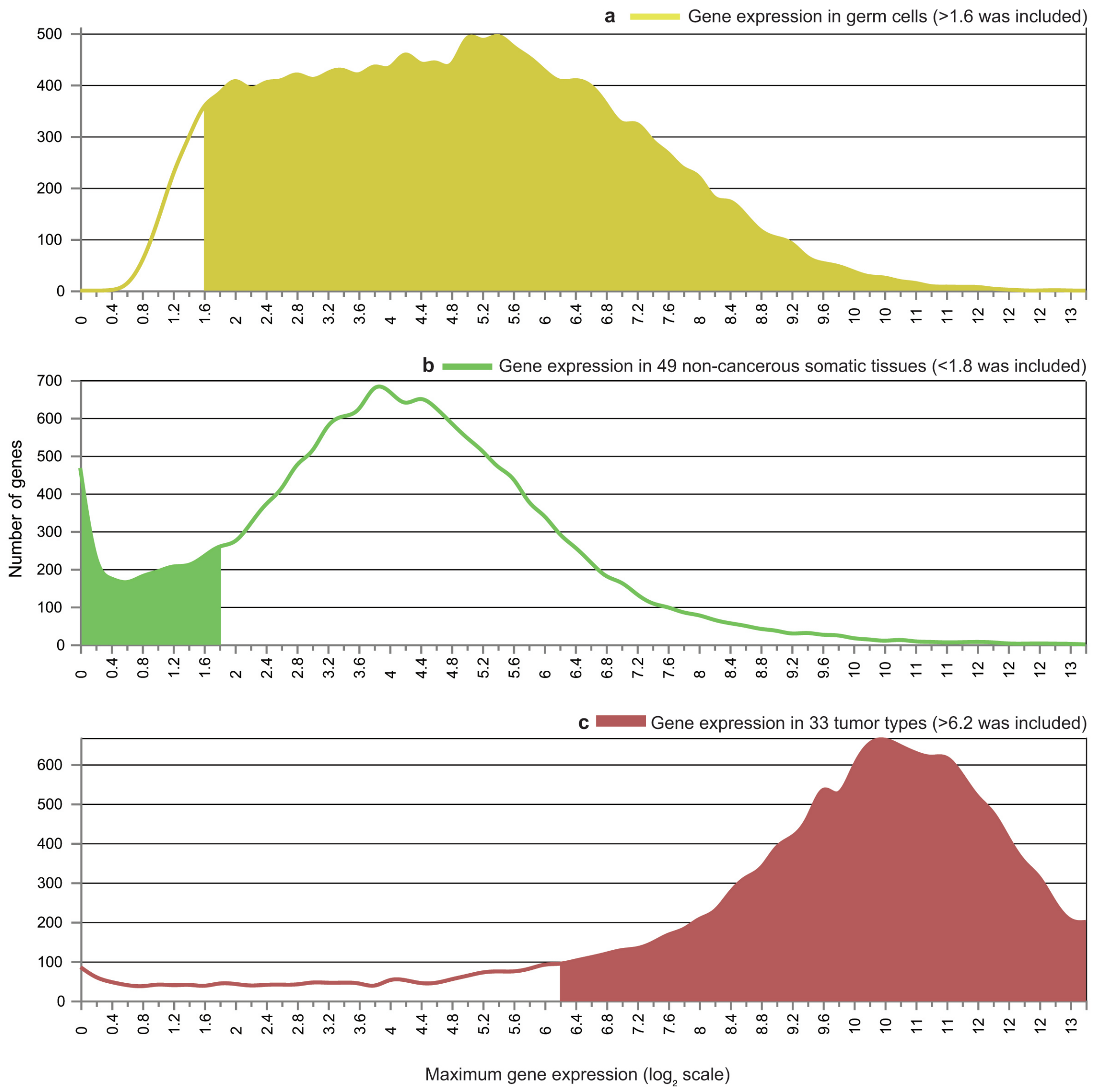

Supplementary Figure 1. Selection of genes based on gene expression in three datasets. From the 16 589 adult male germ cell genes, 1 526 genes were excluded for which no information was available on the expression in either non-cancerous somatic tissues[6] or tumors[7] (**supplementary data 9**). **a**, For gene expression in germ cells, genes with a maximum expression below 1.6 on a $\log_2$ scale were considered background noise and were excluded. **b**, Likewise, in order to only include genes that are exclusive to the male germ cells, genes with an expression over 1.8 on a $\log_2$ scale in any non-cancerous somatic tissue were also excluded. **c**, Finally, we selected for genes with an expression higher than 6.2 on a $\log_2$ scale in at least one of 33 tumor types.
