# Supplementary material for: Massive expression of germ cell-specific genes is a hallmark of cancer and a potential target for novel treatment development
Source: Oncogene. 2018 Jun 15;37(42):5694–700. doi: 10.1038/s41388-018-0357-2 (PMC6193945; doi:10.1038/s41388-018-0357-2)
Supplement: Supplementary file 3 — Supplementary Figure 2 [file 41388_2018_357_MOESM3_ESM.pdf]

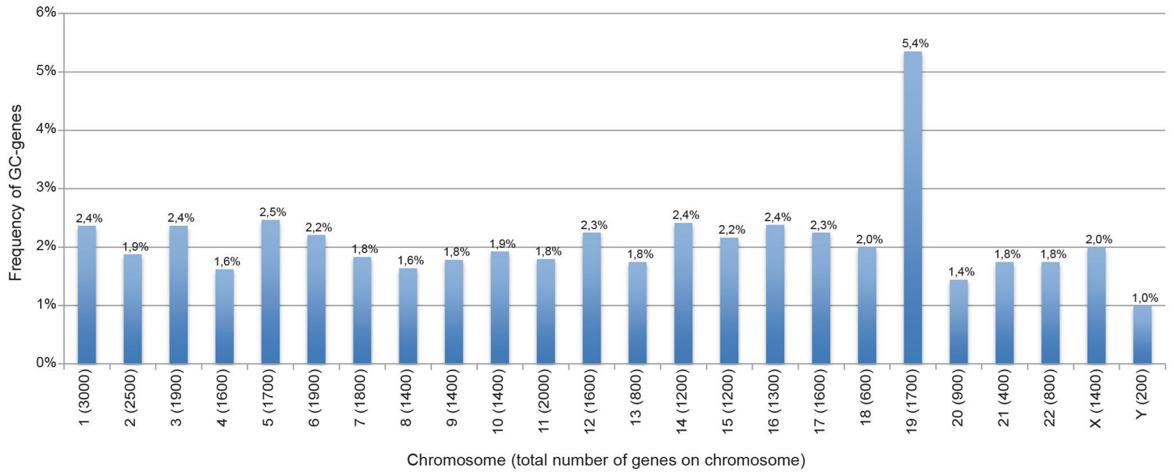

Supplementary Figure 2. GC-genes are not overly expressed from the X-chromosome. Relative occupation of germ cell specific cancer (GC) genes on each chromosome. The percentage shows the number of GC-genes divided by the total number of genes on that chromosome.
